# Identifying patients most likely to benefit from routine post-operative mammogram after breast-conserving surgery

**DOI:** 10.1007/s10549-025-07820-5

**Published:** 2025-10-28

**Authors:** Naama Hermann, Renata Faermann, Keren Grinin, Miri Sklair-Levy, Einav Nili Gal-Yam, Keren Levanon, Tehillah S. Menes, Orit Kaidar-Person, Nora Balint-Lahat, Opher Globus

**Affiliations:** 1 School of Medicine, Faculty of Medical & Health Sciences, Tel Aviv University, Tel Aviv, Israel; 2 Division of Surgery, Sheba Medical Center, Ramat Gan, Israel; 3 Division of Diagnostic Imaging, Sheba Medical Center, Ramat Gan, Israel; 4 The Jusidman Cancer Center, Sheba Medical Center, Ramat Gan, Israel; 5 Breast Radiation Unit, Sheba Medical Center, Ramat Gan, Israel; 6 GROW - School for Oncology and Developmental Biology, Maastricht University, Maastricht, The Netherlands; 7 Institute of Pathology, Sheba Medical Center, Ramat Gan, Israel; 8 School of Medicine, Tel Aviv University, Tel Aviv, Israel

**Keywords:** Routine post-operative mammogram, Residual calcifications, Residual malignancy, Breast conserving Surgery, Post-operative calcifications

## Abstract

**Background:**

Routine post-operative mammograms (RPMs) are performed at some institutions after breast-conserving surgery (BCS) in patients who presented with malignant calcifications in order to rule out residual malignancy. However, their clinical utility and optimal application remain uncertain.

**Aim:**

To evaluate whether patients diagnosed with breast malignancy due to calcifications on mammography benefit from RPMs after BCS.

**Methods:**

After institutional review board approval, we conducted a retrospective cohort study of patients presenting with malignant calcifications on initial screening mammograms who underwent RPMs at our institution between 2018 and 2022. Patients with positive surgical margins or those who underwent imaging for clinical indications were excluded. Imaging findings, pathology results, and clinical characteristics were analyzed to identify factors associated with residual malignancy.

**Results:**

During the study period, 2054 patients underwent BCS, of whom 306 (15%) had a post-operative mammogram within three months of surgery, and 218 fitted the final inclusion category. Suspicious residual calcifications after BCS were identified in 22 of 218 patients (10%), of whom 19 underwent biopsy and 3 proceeded directly to surgery. Residual malignancy was confirmed by biopsy in 9 patients (4%), with a positive predictive value of 41%. Multivariate analysis demonstrated that younger age and the extent of calcifications on preoperative mammograms were independently associated with residual malignancy on RPM.

**Conclusions:**

RPMs were found to be more beneficial for patients aged 50 years or younger, and for patients with extensive calcifications on preoperative mammograms. Tailoring RPM use to these subgroups may improve diagnostic efficiency and reduce unnecessary interventions.

## Introduction

Microcalcification is a key finding in mammography, and is often the presenting sign of breast neoplasia, mainly ductal carcinoma in situ (DCIS) [1]. The indications for routine post-operative mammograms (RPMs) in patients undergoing breast-conserving surgery (BCS) to confirm that calcifications were completely removed during surgery are not well established in current literature or guidelines.  Commonly used international guidelines primarily recommend a baseline mammogram 6–12 months after completing radiotherapy and advocate for personalized surveillance strategies [2, 3]. However, they lack specific recommendations for patients presenting with malignant calcifications. This is especially relevant for patients with pathologically confirmed clear surgical margins, defined as ≥ 2 mm DCIS or no ink on the tumor for invasive carcinoma—criteria generally considered indicative of adequate malignancy removal [4, 5].

Despite the lack of clear guidelines, some institutions routinely perform RPMs in patients with malignant calcifications to ensure complete excision of disease before initiating radiotherapy. The rationale is that undetected residual malignancy may persist even with pathologically clear margins, potentially impacting long-term oncologic outcomes. However, there is no consensus on whether RPM meaningfully alters patient management or improves local recurrence rates. Since pathologically negative surgical margins are generally considered reliable indicators of complete tumor removal, the necessity of additional radiological evaluation remains debated [6, 7]. Implementing RPM is not without its disadvantages, including false-positive findings that may lead to unnecessary biopsies or surgeries, delays in adjuvant therapy, and increased healthcare costs [6].

Given the absence of standardized guidelines, the clinical benefit of RPM remains unclear. There is a need to determine whether certain patient subgroups, are more likely to harbor residual malignancy and therefore derive greater benefit from RPM.

## Methods

We conducted a retrospective cohort study, reviewing all cases of RPMs performed after surgery and before radiotherapy in patients who underwent BCS between January 23, 2018, and May 9, 2022. The study was approved by the institutional review board.

At our institution, the standard approach for patients with malignant calcifications undergoing BCS includes pre-surgical fine-needle localization and bracketing, intraoperative specimen mammography, and a routine pre-radiotherapy mammogram.

### Patient selection criteria

Inclusion criteria:

1. Malignant calcifications on initial diagnostic mammogram.
2. Pathology-confirmed clear surgical margins (≥ 2 mm for DCIS, no ink on tumor for invasive carcinoma).

Exclusion criteria:

1. Post-operative mammogram done for clinical indications (e.g., abnormal physical exam findings, pathology-clinical discordance, or clip localization).
2. Patients whose preoperative mammograms did not demonstrate calcifications.
3. Patients who had residual calcifications more than 2 cm away from the surgical cavity (identified by surgical clips or seroma), as we considered them to be a failure in preoperative workup rather than residual calcification left after the lumpectomy.

Using MDClone software (MDClone, Beer Sheba, Israel), we identified all patients who underwent BCS at our institution during the study period. RPMs were defined as mammograms performed within three months post-surgery in patients who had malignant calcifications on their preoperative mammograms, without other specific clinical indications such as close or positive surgical margins or missing biopsy clips. All preoperative mammograms leading to the initial diagnosis were retrospectively reviewed by a fellowship-trained breast radiologist to assess calcifications’ characteristics, including extent, distribution, and morphology. The largest calcification extent was measured for each case. The radiologist was blinded to RPM images and clinical/surgical outcomes.

All mammograms were performed using the Senographe Pristina Mammography System™ (GE Healthcare, Chicago, IL, USA) and interpreted at the Breast Imaging Unit of the Meirav Center for Women’s Health at Sheba Medical Center. Only the breast undergoing BCS was imaged, using orthogonal mediolateral and craniocaudal views, with magnification views of the surgical bed. RPM findings were classified as suspicious or non-suspicious, and biopsy recommendations were based on original prospective radiology reports, allowing assessment of RPM’s real-world clinical contribution.

Residual malignancy was defined as the presence of DCIS or invasive carcinoma confirmed by vacuum-assisted biopsy or repeated surgical excision. Stereotactic-guided vacuum-assisted biopsies were performed using the Hologic Lorad Prone Breast Biopsy Unit with a 9G needle until 2018, and confirmation of calcification sampling performed in the regular mammography unit. Starting from 2019, the Affirm Prone Biopsy System (Hologic, Inc., Marlborough, Massachusetts) with a 9G needle was used. The latter was coupled with the Hologic Inc. CorLumina system for real time confirmation of calcification sampling.

A comprehensive review of medical records for all patients who underwent RPM was conducted to collect demographic, clinical, radiological, and pathological data. This included patient age, receipt of neoadjuvant systemic treatment (NAST), body mass index (BMI), breast density (BI-RADS classification), tumor characteristics, and biopsy or surgical specimen results for residual suspicious calcifications.

To assess associations between patient demographics, imaging findings, pathological characteristics, and the likelihood of residual calcifications or malignancy, we performed univariate analyses using Fisher’s exact test for categorical variables and either the independent t-test or Mann–Whitney U test for continuous variables, based on distribution. Variables significant in univariate analysis were further evaluated using multivariate logistic regression. Statistical significance was defined as a *p*-value < 0.05.

## Results

**Fig. 1:**
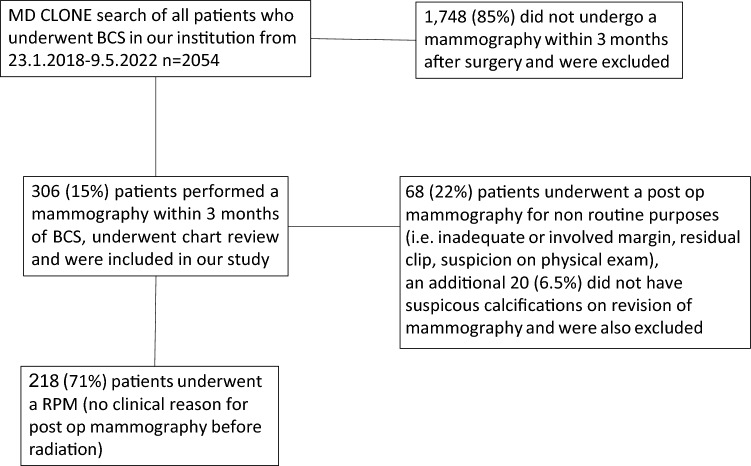
Flow diagram of patient selection

During the study period, 2,054 patients underwent BCS, of whom 306 (15%) had a post-operative mammogram within three months of surgery. A total of 68 patients were excluded as their mammograms were performed for clinical indications and therefore not considered routine. Additional 20 patients were excluded after a preoperative mammogram review showed no evidence of calcifications before surgery, resulting in a final cohort of 218 patients who underwent RPMs. The patient selection process is illustrated in Fig. 1.

The final cohort had a mean age of 57 years (range: 30–88) and a BMI of 26.5 kg/m^2^ (range: 18–46.3). Most patients had dense breast tissue, with 75%  being classified as Breast imaging-reporting and data system (BI-RADS) density C. The most common pathology was pure DCIS, observed in 39% of surgical pathology, followed by invasive ductal carcinoma (IDC) with DCIS (IDC + DCIS) in 38% of the cases. Detailed baseline characteristics of the study cohort are summarized in Table 1.

**Table 1:** General patient characteristics

N	218
Mean Age years(range)	57 (30–88)
Mean BMI (range)	26.5 (18–46.3)
**Breast Density n (%)**	
BI-RADS B	29 (13%)
BI-RADS C	163 (75%)
BI-RADS D	6 (3%)
Missing	20 (9%)
**Lumpectomy pathology n (%)**	
Pure DCIS	84 (39%)
Pure IDC	26 (12%)
IDC + DCIS	83 (38%)
ILC	7 (3%)
pCR	12 (5%)
ypTis	6 (3%)
**ER status n (%)**	
Positive (> 1%)	180 (83%)
Negative (< 1%)	38 (17%)
**Grade n (%)**	
1	28 (13)
2	103 (47)
3	77 (35)
Missing	10 (5)
**Multifocal n (%)**	
Yes	60 (27)
No	158 (73)
**NAST n (%)**	
Yes	44 (20)
No	174 (80)
Mean largest measurement of the calcifications area on pre-surgical mammography (cm) (range)	2.7 (0.1–12)

*BMI* Body Mass Index, *BI-RADS* Breast imaging-reporting and data system, *DCIS* Ductal carcinoma in situ, *IDC* Invasive ductal carcinoma, *ILC* Invasive Lobular carcinoma, *pCR* pathological complete response, *ypTis* pathological complete Response of the invasive component + residual DCIS ,*NAST* Neoadjuvant systemic treatment

**Fig. 2:**
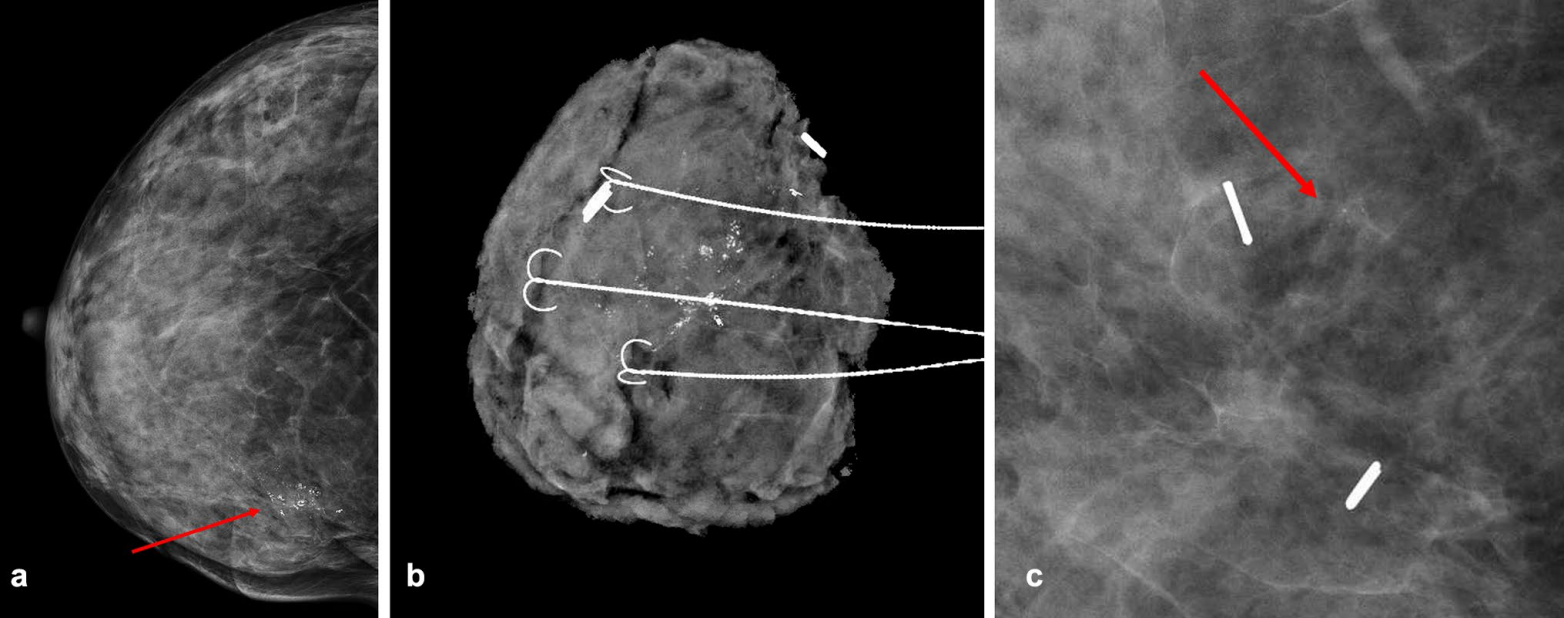
**A** Pre-operative mammogram, CC view, showing a large cluster of suspicious calcifications at the inner part of the right breast. Mammotome biopsy showed DCIS (arrow). **B** Intra-operative X-ray showing excised calcifications. **C** Post-operative ammogram showing residual malignant calcifications in the surgical bed (arrow)

RPM identified residual suspicious calcifications in 22 patients (10%). Of these, 19 (86%) underwent biopsy, while 3 (14%) proceeded directly to surgery at the surgeon’s discretion. Malignancy was confirmed by biopsy or surgical pathology in 9 of the 22 patients with findings on RPM (41%), all of which were DCIS. Among these 9 patients, 5 underwent re-lumpectomy, 3 underwent mastectomy, and 1 was lost to follow-up. Of the 3 patients who proceeded directly to surgery, 2 had residual DCIS and one had a benign lesion. Mammographic findings and biopsy results of the residual calcifications are summarized in Table 2. Examples of preoperative mammogram, intraoperative specimen mammogram, and RPM showing residual malignant calcification are shown in Fig. 2.

**Table 2:** Pathology Results of suspicious residual calcifications identified in 22 out of 218 routine post-operative mammograms, Classified according to ACR BI-RADS® Atlas, 5th Edition [8]

Pathology Diagnosis	n (%)	Calcification Characteristics
**Malignant Lesions:**		
DCIS	9 (40.9%)	Fine pleomorphic (5), amorphous (4)
**Benign lesions:**		
Fat Necrosis	3 (13.6%)	Diffuse punctate (2), coarse heterogeneous (1)
Fibrocystic Changes	1 (4.5%)	Diffuse punctate
Other Benign Lesions	2 (9.1%)	Coarse heterogeneous (1), amorphous (1)
**High Risk Lesions**:		
ADH	2 (9.1%)	Amorphous (1), fine pleomorphic (1)
ALH	1 (4.5%)	Amorphous
LCIS	1 (4.5%)	Fine pleomorphic
**Other Lesions**		
Papillary Lesion	2 (9.1%)	Dystrophic (1), punctate (1)
UDH	1 (4.5%)	Coarse heterogeneous

*DCIS* Ductal Carcinoma in Situ, *ADH* Atypical Ductal Hyperplasia, *LCIS* Lobular Carcinoma in situ, *ALH* Atypical Lobular Hyperplasia, *UDH* Usual Ductal Hyperplasia

Univariate analysis identified two key predictors of residual malignancy: younger age and greater calcification extent at presentation. Patients aged 50 years or younger had significantly higher rates of residual suspicious calcifications (16.7% vs. 6.8%, *p* = 0.03) and residual malignancy (11.1% vs. 0.7%, *p* = 0.0007) in comparison to patients over 50. Similarly, patients with more extensive calcifications (area > 3 cm) were more likely to have residual calcifications (16.9% vs. 6.8%, *p* = 0.02) and residual malignancy (9.9% vs. 1.4%, *p* = 0.006). Pure DCIS on the lumpectomy pathology was significantly associated with residual calcifications (15.9% vs. 6.2%, *p* = 0.02), and showed a trend toward residual malignancy which was not significant (6.8% vs. 2.3% *p* = 0.16).

No significant associations were observed between residual calcifications or malignancy and other clinicopathological factors, including BMI, breast density, tumor grade, focality, estrogen receptor (ER) status, HER2 status, and receipt of NAST.

In multivariate logistic regression, younger age and greater calcification extent remained independent predictors of residual malignancy. The likelihood of residual malignancy decreased with increasing age (OR: 0.93 per year, 95% CI: 0.87–0.99, *p* = 0.04), while each additional centimeter of calcification extent was associated with an increased risk of residual DCIS (OR: 1.3 per cm, 95% CI: 1.07–1.7, *p* = 0.01).

## Discussion

The findings of this study suggest that RPM can provide meaningful additional diagnostic value in patients with malignant calcifications who have undergone BCS with clear surgical margins. In our cohort, the positive predictive value (PPV) for biopsy-indicated cases was 41%, indicating that a significant proportion of patients with residual calcifications on RPM also had residual disease. These results support the potential role of RPM in detecting residual malignancy beyond what can be ensured by surgical margins alone.

Previous studies have reported that the prevalence of residual calcifications on post-operative mammograms ranges from 2 to 24%, with 2% to 9% of patients found to have residual malignancy based solely on findings from pre-radiotherapy mammography [7, 10, 11]. Our findings align with these results, as 10% of RPMs revealed residual calcifications, and 4% of patients in our cohort were found to have residual malignancy.

In our cohort, a calcification extent greater than 3 cm at diagnosis was significantly associated with both residual calcifications on RPM and residual malignancy on pathology. Rodrigues-Duarte et al. also found calcifications extent larger than 5 cm to be associated with residual calcifications [11]. Interestingly, they did not find the extent of calcifications to be associated with a greater percentage of residual malignancy. Residual calcifications were also more common when the lumpectomy pathology was pure DCIS, a finding consistent with a previous paper by Pires et al. [9]. We hypothesize that larger lesions and DCIS (which is more commonly non-palpable) may be more challenging to localize and bracket preoperatively, as well as to identify intraoperatively. Additionally, the desire to preserve cosmetic outcomes in cases of larger lesions may lead surgeons to take smaller surgical margins, potentially increasing the likelihood of residual disease.

Similar to Azam et al. [1], menopausal status played a significant role in finding residual malignant calcification. Residual calcifications identified on RPM in younger patients were significantly more likely to represent residual malignancy, with two thirds of calcifications in patients aged 50 years or younger found to be malignant. While there is no direct evidence that RPM impacts local recurrence rates [7], younger patients are known to have a higher risk of local recurrence after BCS for DCIS and therefore may derive particular benefit from RPM [12].

In contrast, 90% of calcifications in patients over 50 years were benign. The high rate of false-positive findings in older patients highlights the potential drawbacks of RPM in this population.

Performing RPM can impose substantial financial and emotional burdens on both patients and the healthcare system [13]. Its drawbacks include a low diagnostic yield due to post-surgical tissue changes and false-positive findings that may lead to unnecessary interventions, such as biopsies or surgeries.

Nevertheless, RPM may play a role in distinguishing early local recurrence from residual disease. Identifying residual disease before radiotherapy allows for additional surgery- either breast-conserving or mastectomy- without significantly impacting adjuvant treatment plans. Conversely, if the initial mammogram is performed only after radiotherapy, any detected disease is more likely to be classified as an early recurrence due to local treatment failure rather than residual disease. This distinction often necessitates mastectomy and may also prompt consideration of more extensive systemic treatment.

Our findings should be interpreted in light of the known multifocal nature of breast cancer and the established role of radiotherapy in achieving local control. Holland et al. [14] demonstrated that up to 63% of early stage breast cancers harbor additional foci of disease, with 43% located more than 2 cm from the index tumor. This underscores the potential for residual microscopic disease, even when both surgical margins and routine post-operative mammography (RPM) are clear. In this context, post-operative radiotherapy remains essential to address occult foci and reduce the risk of local recurrence. Long-term data from the NSABP B-06 trial by Fisher et al. [15] showed that the addition of radiotherapy after lumpectomy significantly reduced the rate of ipsilateral breast tumor recurrence (14.3% with radiation vs. 39.2% without; *p* < 0.001), although overall survival remained comparable across treatment groups. All patients in our cohort received adjuvant radiotherapy. Our findings support the complementary role of RPM- not as a substitute for radiotherapy, which is aimed at treating additional, non-detectable multifocal lesions- but as an additional tool to identify radiographically visible residual disease that can be addressed surgically prior to radiation.

This study has several limitations, including its retrospective design, relatively small sample size, and single-center setting which may limit generalizability. In addition, we did not assess long-term outcomes such as local recurrence, nor can we determine whether the residual malignant foci identified would have been adequately treated with radiotherapy alone. These limitations highlight the need for prospective, multi-center studies with long-term follow-up to better evaluate the oncologic impact of routine post-operative mammography after breast-conserving surgery. However, we believe it highlights an important and somewhat overlooked post-operative diagnostic practice, by better defining the subset of patients most likely to benefit from it.

Future prospective studies with larger, multi-center cohorts as well as long-term follow-up are needed to validate these findings and refine patient selection criteria for RPM.

## Conclusion

Our study suggests that RPM after BCS should be strongly considered in younger patients (< 50 years) with extensive malignant calcifications (> 3 cm) at diagnosis, even when surgical margins are clear. However, our results do not support routine RPM in older patients with malignant calcifications who underwent BCS with clear surgical margins.

## Data Availability

No datasets were generated or analysed during the current study.
